# Temporal trends and projected mortality of myocardial infarction and heart failure in the United States, 1999–2035: a CDC WONDER analysis

**DOI:** 10.3389/fcvm.2026.1772415

**Published:** 2026-07-09

**Authors:** Muhammad Hussain Azam, Muhammad Hassan Azam, Divesh Sunil Sachdev, Hasibullah Aminpoor, Taha Ahmed, Talha Aamir, Muhammad Tayyab Azam, Nazila Dalir, Faizan Ahmed

**Affiliations:** 1 Department of Medicine, Dow University of Health Sciences, Karachi, Pakistan; 2 Faculty of Medicine, Kabul University of Medical Sciences “Abu Ali Ibn Sina”, Kabul, Afghanistan; 3 School of medicine, St. George's University, St. George's, Grenada, West Indies; 4 Department of Internal Medicine, Jersey shore University Medical Centre, Neptune, New Jersey, United States

**Keywords:** CDC WONDER, heart failure, myocardial infarction, reseach, trend analysis

## Abstract

**Background:**

Myocardial infarction (MI) and heart failure (HF) remain major contributors to cardiovascular mortality in the United States. This study analyzed national MI- and HF-related mortality trends from 1999 to 2024 and projected rates through 2035.

**Methods:**

Mortality data for adults aged ≥25 years with MI- and HF-related deaths (ICD-10 codes I21–I22 and I50) were obtained from CDC WONDER. Age-adjusted mortality rates (AAMRs), standardized to the 2000 U.S. population, were calculated. Temporal trends were assessed using Joinpoint regression to estimate annual percent change (APC) and average annual percent change (AAPC). Forecasts through 2035 were generated using Auto-ARIMA and Prophet models.

**Results:**

From 1999 to 2024, 552,835 myocardial infarction (MI)- and heart failure (HF)-related deaths were recorded in the United States. The mean age-adjusted mortality rate (AAMR) across the study period was 9.73 per 100,000 population. The national AAMR declined from 15.32 in 1999 to 7.55 in 2024, with a significant decrease from 1999 to 2012 (APC = −5.39%, *p* < 0.000001) followed by relative stabilization thereafter. By 2035, the national AAMR is projected to increase modestly to 8.41 (95% CI: 7.46–9.31). Mortality rates were consistently higher in men than women (mean AAMR: 12.33 vs. 7.87 per 100,000) and in non-Hispanic Black individuals compared with non-Hispanic White and Hispanic individuals (10.59 vs. 9.92 vs. 7.70 per 100,000). Regional variation persisted, with the South demonstrating the highest mortality burden (mean AAMR: 10.52; projected 2035: 10.25) and the Northeast the lowest (mean AAMR: 8.34; projected 2035: 3.75). Mortality remained highest among adults aged ≥65 years, while increasing among adults aged 25–44 years after 2010. Rural populations consistently exhibited higher mortality than urban populations, with projected 2035 AAMRs of 13.94 and 7.73 per 100,000, respectively.

**Conclusions:**

Although MI- and HF-related mortality declined substantially from 1999 to 2024, progress slowed after 2012, with persistent demographic and geographic disparities across age, race, region, and rurality. Younger adults, rural populations, and non-Hispanic Black individuals continue to experience disproportionately higher mortality burdens, underscoring the need for equitable prevention strategies and improved cardiovascular care access

## Introduction

Cardiovascular disease (CVD) continues to exact an immense toll on global health, remaining the world's leading cause of mortality and disability and accounting for 19.8 million deaths in 2022 alone (1). Among its major manifestations, myocardial infarction (MI) is defined as acute coronary artery occlusion resulting in myocardial necrosis, while heart failure (HF) is a complex clinical syndrome characterized by structural or functional impairment of ventricular filling or ejection of blood (2). These two conditions are closely linked both pathophysiologically and epidemiologically. MI often precipitates HF through ischemia-induced cardiomyocyte death, which initiates adverse ventricular remodeling (AVR) characterized by left ventricular dilation, hypertrophy, and myocardial fibrosis (3). This remodeling process is mediated by mechanical stress, persistent inflammation, and activation of the sympathetic nervous system (SNS) and renin-angiotensin-aldosterone system (RAAS), ultimately leading to progressive systolic and diastolic dysfunction (4).

In addition, ischemia-reperfusion injury and microvascular obstruction contribute to infarct expansion and impaired healing, while mitochondrial dysfunction and oxidative stress promote ongoing cardiomyocyte apoptosis and disrupt metabolic homeostasis (4, 5). These maladaptive changes create a substrate for ventricular arrhythmias and sudden cardiac death, which account for about 30%–40% mortality in post-MI HF (6). The remaining mortality burden arises from progressive pump failure, characterized by declining cardiac output and chronic end-organ hypoperfusion (7). Together, these mechanisms help explain the persistent long-term mortality burden associated with MI and HF (8).

Recent national analytical studies report divergent patterns in the historical trends of MI and HF mortality. From 1999 to 2012, age-adjusted mortality rates (AAMRs) for acute MI and HF declined substantially, followed by a plateau or upturn from 2012 to 2019 among both older and younger adults (9, 10). This upturn coincides with rising obesity and diabetes prevalence, which accelerate atherosclerotic progression and myocardial dysfunction, significantly amplifying the lifetime risk of heart failure (11). The COVID-19 pandemic further exacerbated these shifts from 2020 to 2022, contributing to excess CVD mortality through direct cardiac injury and thrombotic complications (12, 13). Provisional data through 2023 indicate sustained elevations, with heart disease reclaiming primacy as the leading cause of death (14).

Demographic and geographic evidence also reveal inequities in MI- and HF-related outcomes. Men, especially non-Hispanic Black or African American persons, exhibit higher AAMRs than women across all age groups (9, 10). Furthermore, Southern and Midwestern states experience a disproportionately higher burden compared with the Northeast and the West, correlating with higher obesity, inactivity, and poverty rates in non-metropolitan areas (15).

Despite the clinical and policy importance of both conditions, most observational trend reports and prior projections treat MI and HF separately. The American Heart Association (AHA) and major forecasting efforts have emphasized the need for integrated burden projections (16); yet limited evidence combines multi-decadal empirical trends for MI and HF, estimates transition probabilities, and produces transparent forecasts to 2035. To address these gaps, we developed a comprehensive study leveraging CDC WONDER multiple-cause mortality data for MI and HF to quantify empirical patterns, model transition probabilities from 1999 through 2024, and project incidence, prevalence, and mortality to 2035 under baseline and alternative mortality risk-factor scenarios.

## Methods

### Study settings and population

This longitudinal observational study analyzed national mortality related to myocardial infarction (MI) and heart failure (HF) among adults aged ≥25 years in the United States using data from the Centers for Disease Control and Prevention (CDC) Wide-ranging Online Data for Epidemiologic Research (WONDER) database. Mortality data were extracted for the years 1999–2024 through death certificates compiled by the National Vital Statistics System (NVSS). Cases were identified using the International Statistical Classification of Diseases and Related Health Problems, 10th Revision (ICD-10) codes of MI (I21 for acute MI, I22 for subsequent MI) and HF (I50), in accordance with previous national surveillance studies (17, 18). This research was exempt from Institutional Review Board (IRB) review as it relied solely on publicly available, deidentified data. All procedures adhered to the Strengthening the Reporting of Observational Studies in Epidemiology (STROBE) guidelines to ensure transparency, reproducibility, and methodological rigor (19).

### Data abstraction

Mortality counts and population estimates were stratified by calendar year, sex, race/ethnicity, age group, Census region, and urban–rural status. Sex was categorized as male or female. Race/ethnicity was recorded on death certificates and grouped as Hispanic, non-Hispanic (NH) White, NH Black, and NH Asian or Pacific Islander. Age was grouped into three strata: 25–44 years (younger adults), 45–64 years (middle-aged adults), and ≥65 years (older adults). Urbanicity was defined according to the 2013 National Center for Health Statistics (NCHS) Urban–Rural Classification Scheme, which classifies counties as urban (population ≥50,000) or rural (population <50,000) (20). Census regions were delineated as Northeast, Midwest, South, and West. Additionally, a sensitivity analysis was conducted by defining heart failure as a multiple cause of death and myocardial infarction as the underlying cause of death to further evaluate their bidirectional contribution to mortality trends. However, when heart failure was specified as the underlying cause of death, the available data were sparse and incomplete across multiple years, resulting in unstable estimates that precluded reliable trend analysis. Therefore, this reverse configuration was not included in the final analyses. All data were obtained under the provisions of the Public Health Service Act [42 U.S.C. 242m(d)] and used exclusively for statistical purposes. No individual identification was attempted, and confidentiality was maintained under the supervision of the National Center for Health Statistics Confidentiality Officer.

### Statistical analysis

Crude and age-adjusted mortality rates (AAMRs) per 100,000 population were computed annually by year, sex, race/ethnicity, and census region, standardized to the 2000 U.S. standard population using the direct method (21). Temporal trends were analyzed using the Joinpoint Regression Program (National Cancer Institute) to quantify the annual percent change (APC) and 95% confidence intervals for AAMRs (22). Average annual percent change (AAPC) was calculated as a weighted average of APC estimates across all identified Joinpoint segments, weighted according to the duration of each segment, and was used to summarize the overall temporal trend over the study period. The slope of each trend was categorized as increasing or decreasing based on deviation from zero in APC, with statistical significance determined using a two-tailed *t*-test. A *p*-value less than 0.05 denotes statistical significance. All data analyses adhered to CDC data-use and confidentiality requirements. Combined analyses of MI- and HF-related mortality were conducted to estimate the overall cardiovascular mortality burden.

### Time series forecasting

Age-adjusted mortality rates for MI and HF from 1999 to 2024 were modeled to forecast trends through 2035. Two complementary forecasting approaches, auto-regressive integrated moving average [Auto-ARIMA (specifying automatically selected *p*, *d*, *q* parameters based on AIC minimization)] and Prophet (Prophet model with default additive trend specification, yearly seasonality enabled, and no external regressors unless specified), were employed using R software (R Foundation for Statistical Computing, Vienna, Austria) (23). The models were trained on data from 1999 to 2020, with performance validated using rolling one-year-ahead forecasts for 2021–2024 to ensure predictive robustness. At each iteration (*t*), the model was refitted using data through year t to forecast year *t* + 1. Model performance was assessed using the root mean squared error (RMSE) on the natural scale, and the optimal model (lowest RMSE) was selected. The final RMSE values for both Auto-ARIMA and Prophet models were reported and compared to determine the superior forecasting approach. This model was then retrained on the full 1999–2024 dataset to project AAMRs for 2025–2035 with 95% prediction intervals to reflect forecast uncertainty.

Here is the full revised Results section with the last lines updated to show the best-performing model and both RMSE values clearly.

## Results

### National trends and overall mortality (1999–2024; projections to 2035)

During 1999–2024, a total of 552,835 deaths were attributed to myocardial infarction (MI) with heart failure (HF) as a contributing cause in the United States. The age-adjusted mortality rate (AAMR) declined from 15.32 per 100,000 in 1999 to 7.55 per 100,000 in 2024 (Table 1, Supplementary Table 3). A significant decline was observed from 1999 to 2012 (APC: −5.39%, 95% CI: −5.98 to −4.89; *p* < 0.000001), followed by a stabilization from 2012 to 2024 (APC: 0.35%, 95% CI: −0.32 to 1.18; *p* = 0.259548) (Supplementary Table 2). The mean AAMR across the study period was 9.73 per 100,000. The Prophet model (RMSE: 0.75) outperformed the ARIMA model (RMSE: 0.86). By 2035, the Prophet model projected an overall AAMR of 8.41 per 100,000 (95% CI: 7.46–9.31) (Supplementary Table 8).

**Table 1:** Age-adjusted mortality rate (AAMR) and average annual percent change (AAPC) for MI- and HF-related deaths among U.S. adults aged ≥25 years, 1999–2024.

Demographics	Deaths	Population	Overall Age-Adjusted Mortality Rate per 100,000 (95% CI)	AAPC (95% CI)
Overall	552,835	5,398,746,349	9.73 (9.39–9.87)	−2.68* (−2.90 to −2.45)
Sex				
Male	283,557	2,603,935,168	12.33 (11.90–12.76)	−2.33* (−2.51 to −2.08)
Female	269,278	2,790,725,856	7.87 (7.52–8.22)	−3.16* (−3.39 to −2.92)
Race				
NH American Indian or Alaska Native	2,920	39,535,264	10.07 (9.49–10.65)	−1.71* (−2.76 to −0.43)
NH Black	53,733	630,247,138	10.59 (10.40–10.78)	−2.12* (−2.41 to −1.82)
NH White	454,292	3,673,327,998	9.92 (9.78–10.06)	−2.64* (−2.86 to −2.40)
Hispanic	30,662	740,627,884	7.70 (7.52–7.88)	−2.78* (−3.09 to −2.47)
Age				
Younger adults (25–44)	4,883	2,209,338,799	0.22 (0.20–0.24)	1.72* (0.66 to 3.08)
Middle-aged adults (45–64)	66,296	2,024,706,033	3.29 (3.23–3.35)	−0.69* (−0.98 to −0.35)
Older adults (65–85+)	481,656	1,160,616,192	43.52 (42.97–44.07)	−3.02* (−3.24 to −2.81)
Census				
South	215,751	2,005,599,653	10.52 (10.37–10.67)	−2.37* (−2.61 to −2.12)
West	110,437	1,242,121,877	9.10 (8.92–9.28)	−2.16* (−2.45 to −1.86)
Midwest	131,620	1,158,311,489	10.19 (10.01–10.37)	−2.94* (−3.11 to −2.69)
Northeast	95,027	988,628,005	8.34 (8.18–8.50)	−3.61* (−3.85 to −3.38)
Urbanization				
Rural	121,080	678,634,169	14.55 (14.16–14.93)	−2.35* (−2.59 to −2.12)
Urban	342,721	3,795,213,822	9.04 (8.90–9.18)	−3.30* (−3.43 to −3.16)

* represent statistical significance (*p*-value <0.005).

### Sex-specific mortality trends (1999–2024; projections to 2035)

**Figure 1:**
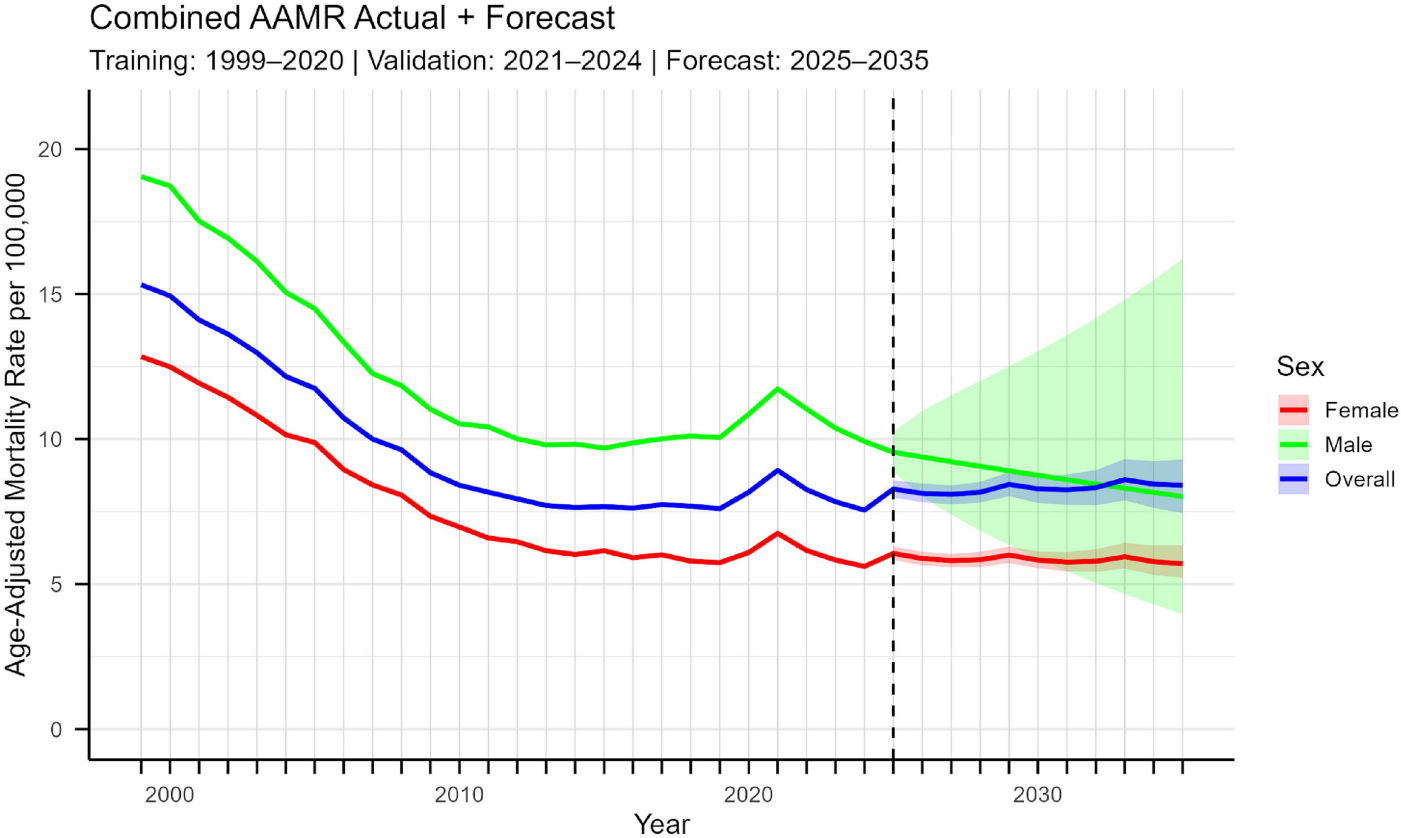
Age-adjusted mortality rates for MI- and HF-related deaths among adults aged ≥25 years, stratified by sex and overall, per 100,000 population, with projections to 2035.

Men consistently exhibited higher mortality than women throughout the study period. Between 1999 and 2024, AAMR declined from 19.05 to 9.92 per 100,000 in men and from 12.84 to 5.61 per 100,000 in women (Supplementary Table 3). The mean AAMR over the study period was 12.33 per 100,000 in men and 7.87 per 100,000 in women. Men experienced a significant decline from 1999 to 2012 (APC: −5.27%, 95% CI: −6.00 to −4.67; *p* < 0.000001), followed by a modest increase from 2012 to 2024 (APC: 0.94%, 95% CI: 0.22–1.84; *p* = 0.012398) (Supplementary Table 2). In contrast, women showed a significant decline from 1999 to 2013 (APC: −5.49%, 95% CI: −6.03 to −5.03; *p* < 0.000001), followed by a period of stabilization from 2013 to 2024 (APC: −0.11%, 95% CI: −0.90 to 0.86; *p* = 0.783443). By 2035, the projected AAMR is 8.02 per 100,000 (95% CI: 3.97–16.22) in men and 5.70 per 100,000 (95% CI: 5.22–6.34) in women (Supplementary Table 8). For men, the ARIMA model (RMSE: 0.93) outperformed the Prophet model (RMSE: 1.04), whereas for women, the Prophet model (RMSE: 0.58) outperformed the ARIMA model (RMSE: 0.74) (Figure 1).

### Racial and ethnic disparities in mortality (1999–2024; projections to 2035)

**Figure 2:**
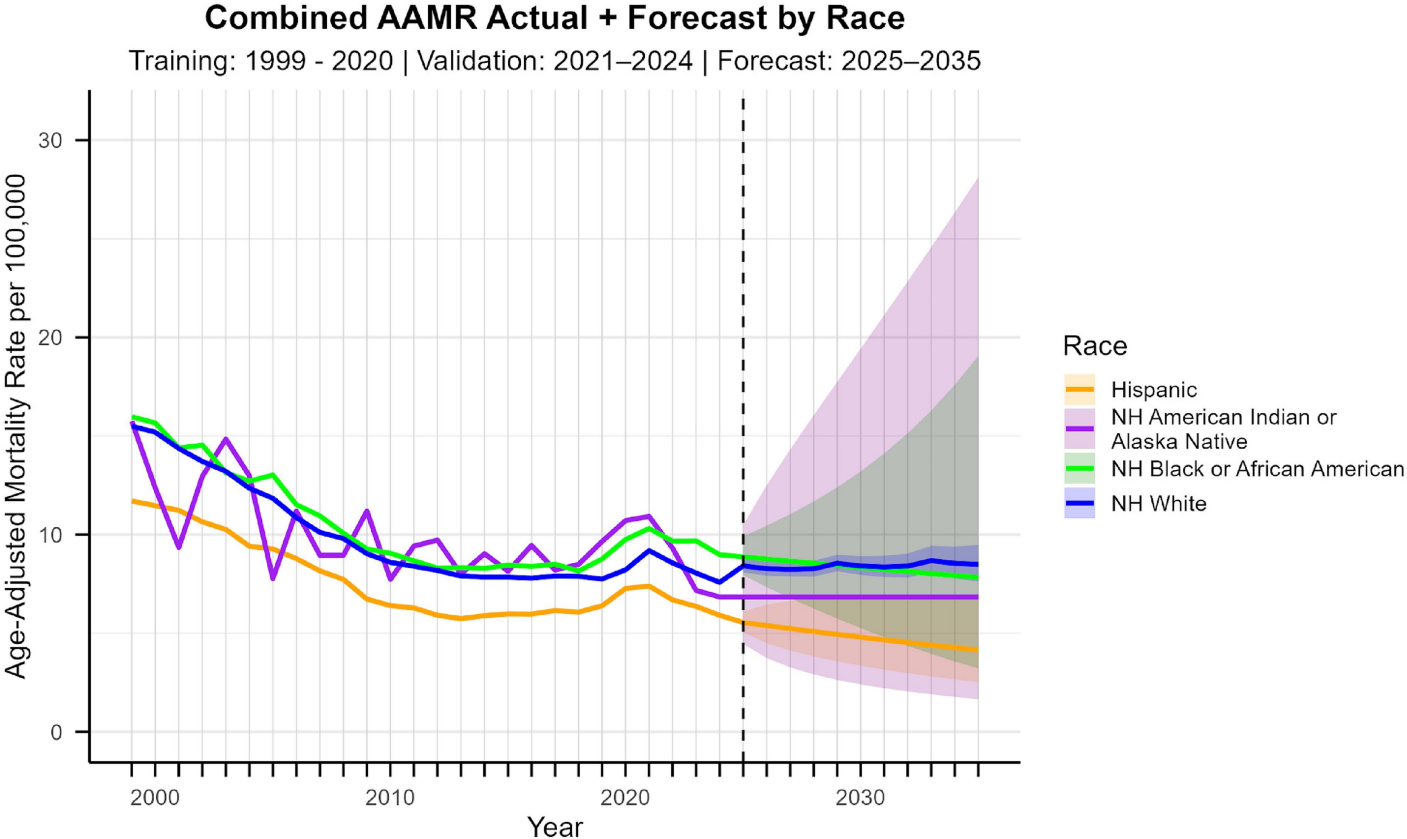
Age-adjusted mortality rates for MI- and HF-related deaths among adults aged ≥25 years, stratified by race/ethnicity, per 100,000 population, with projections to 2035.

Substantial racial and ethnic disparities were observed throughout the study period. The mean AAMR was highest among non-Hispanic Black individuals at 10.59 per 100,000, followed by non-Hispanic American Indian or Alaska Native individuals at 10.07 per 100,000, non-Hispanic White individuals at 9.92 per 100,000, and Hispanic individuals at 7.70 per 100,000 (Supplementary Table 4). Non-Hispanic Black individuals consistently exhibited the highest mortality burden, while Hispanic individuals had the lowest rates throughout the study period. Non-Hispanic Black individuals showed a significant decline from 1999 to 2012 (APC: −5.30%, *p* < 0.000001), followed by a significant increase from 2012 to 2024 (APC: 1.44%, *p* = 0.0004) (Supplementary Table 2). Hispanic individuals demonstrated a decline from 1999 to 2014 (APC: −5.28%, *p* < 0.000001), followed by an increase from 2014 to 2020 (APC: 4.52%, *p* = 0.003199), and a subsequent decline from 2020 to 2024 (APC: −3.84%, *p* = 0.003199). Non-Hispanic White individuals showed a decline from 1999 to 2012 (APC: −5.32%, *p* < 0.000001), followed by stabilization from 2012 to 2024 (APC: 0.36%, *p* = 0.261148). Non-Hispanic American Indian or Alaska Native individuals demonstrated an overall gradual decline over the study period (APC: −1.71%, *p* = 0.010398). By 2035, projected AAMRs are 4.15 per 100,000 (95% CI: 2.53–6.82) in Hispanic individuals, 7.83 per 100,000 (95% CI: 3.21–19.06) in non-Hispanic Black individuals, 8.49 per 100,000 (95% CI: 7.63–9.47) in non-Hispanic White individuals, and 6.84 per 100,000 (95% CI: 1.66–28.13) in non-Hispanic American Indian or Alaska Native individuals (Supplementary Table 9). For Hispanic individuals, the ARIMA model (RMSE: 0.60) outperformed the Prophet model (RMSE: 0.81); for non-Hispanic Black individuals, the ARIMA model (RMSE: 0.71) outperformed the Prophet model (RMSE: 0.96); for non-Hispanic White individuals, the Prophet model (RMSE: 0.85) outperformed the ARIMA model (RMSE: 1.01); and for non-Hispanic American Indian or Alaska Native individuals, the ARIMA model (RMSE: 1.66) outperformed the Prophet model (RMSE: 2.30) (Figure 2).

### Regional differences in mortality (1999–2024; projections to 2035)

**Figure 3:**
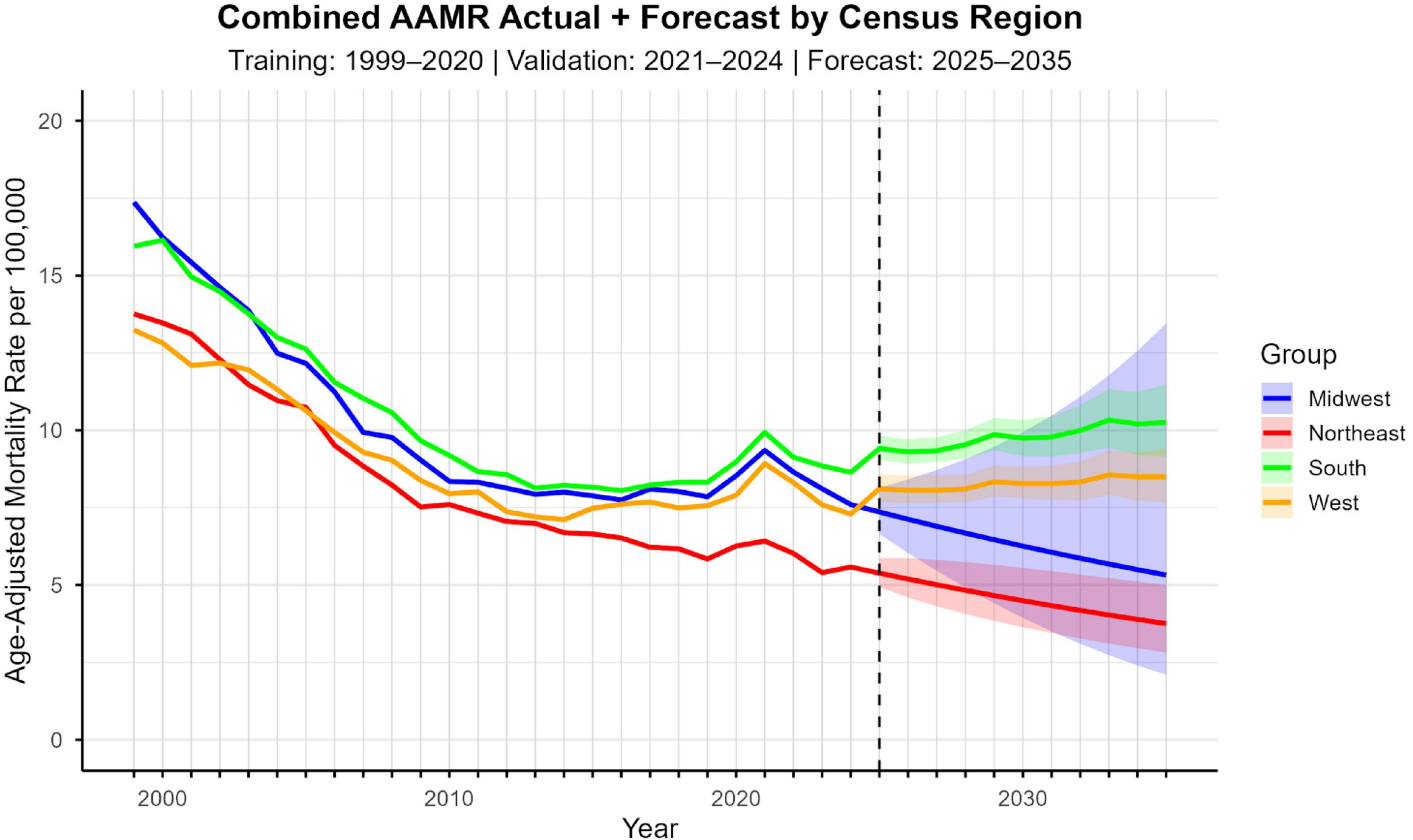
Age-adjusted mortality rates for MI- and HF-related deaths among adults aged ≥25 years, stratified by U.S. Census region, per 100,000 population, with projections to 2035.

Regional variation in mortality was observed across the United States. The mean AAMR was highest in the South at 10.52 per 100,000, followed by the Midwest at 10.19 per 100,000, the West at 9.10 per 100,000, and the Northeast at 8.34 per 100,000 (Supplementary Table 5). All regions demonstrated declining trends until approximately 2012–2013, followed by stabilization or modest increases depending on region. In the South, mortality significantly increased after 2013 (APC: 1.23%, *p* = 0.004399), whereas the Northeast and Midwest showed post-2012 stabilization, and the West demonstrated no significant post-2012 change (Supplementary Table 2). By 2035, projected AAMRs are 10.25 per 100,000 (95% CI: 9.15–11.48) in the South, 8.50 per 100,000 (95% CI: 7.66–9.40) in the West, 5.32 per 100,000 (95% CI: 2.10–13.46) in the Midwest, and 3.75 per 100,000 (95% CI: 2.82–4.99) in the Northeast (Supplementary Table 10). For the Midwest, the ARIMA model (RMSE: 0.83) outperformed the Prophet model (RMSE: 0.86); for the Northeast, the ARIMA model (RMSE: 0.37) outperformed the Prophet model (RMSE: 0.41); for the South, the Prophet model (RMSE: 0.88) outperformed the ARIMA model (RMSE: 1.17); and for the West, the Prophet model (RMSE: 0.88) slightly outperformed the ARIMA model (RMSE: 0.89) (Figure 3).

### Age-stratified mortality trends (1999–2024; projections to 2035)

**Figure 4:**
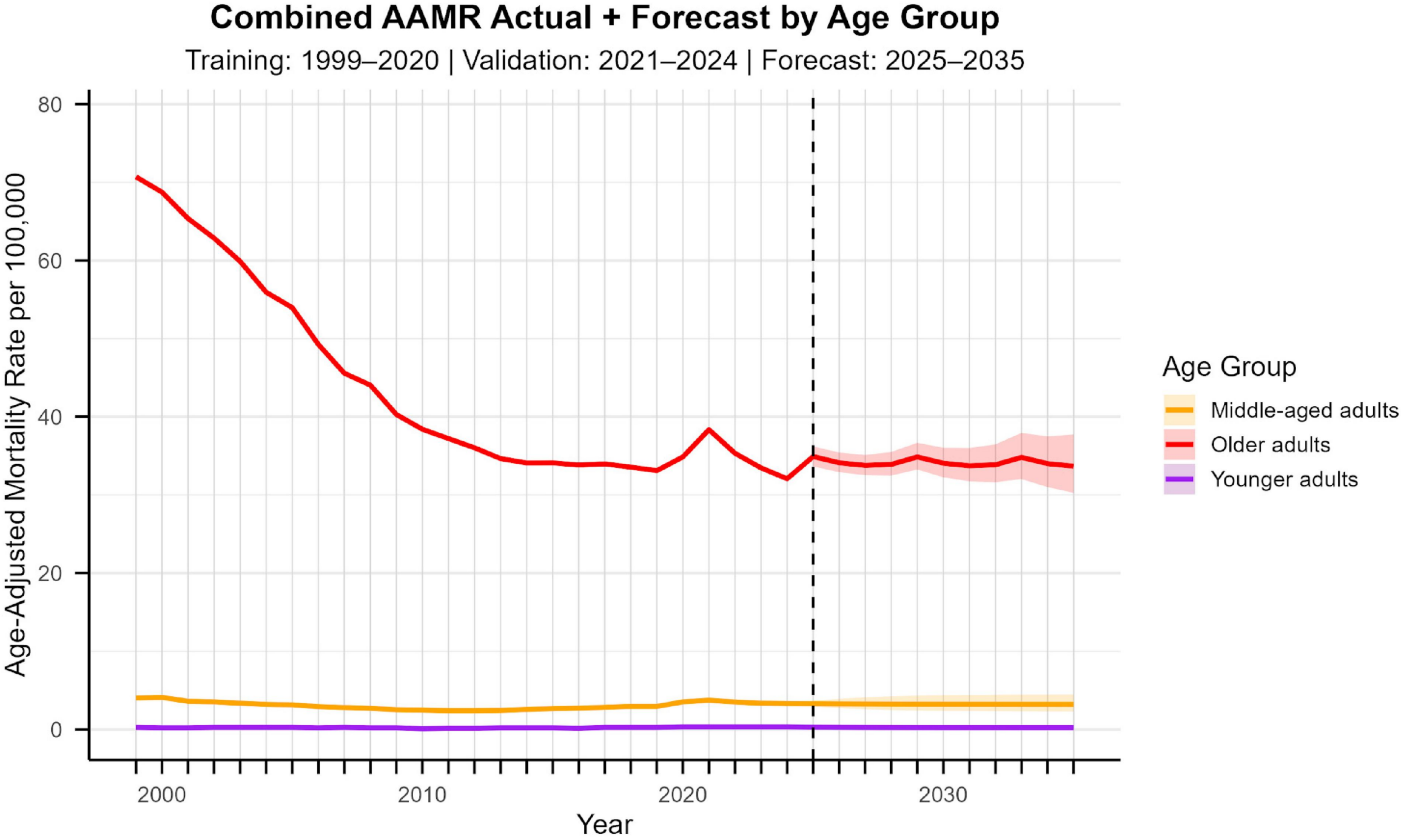
Age-adjusted mortality rates for MI- and HF-related deaths among adults aged ≥25 years, stratified by age group, per 100,000 population, with projections to 2035.

Marked age-related differences were observed in mortality burden. The mean AAMR was 43.52 per 100,000 among adults aged ≥65 years, 3.29 per 100,000 among those aged 45–64 years, and 0.22 per 100,000 among those aged 25–44 years (Supplementary Table 6). Mortality was highest in older adults throughout the study period. Among adults aged ≥65 years, mortality declined significantly from 1999 to 2012 (APC: −5.56%, *p* < 0.000001), followed by stabilization thereafter (APC: −0.20%, *p* = 0.544691). Among adults aged 45–64 years, mortality declined until 2012, increased significantly from 2012 to 2020 (APC: 5.30%, *p* = 0.031994), and then stabilized. Among adults aged 25–44 years, mortality increased significantly from 2010 onwards (APC: 6.31%, *p* = 0.023995) (Supplementary Table 2). By 2035, projected AAMRs are 33.68 per 100,000 (95% CI: 30.27–37.74) in adults aged ≥65 years, 3.21 per 100,000 (95% CI: 2.29–4.49) in those aged 45–64 years, and 0.24 per 100,000 (95% CI: 0.14–0.40) in those aged 25–44 years (Supplementary Table 11). For the 25–44 age group, the ARIMA model (RMSE: 0.05) outperformed the Prophet model (RMSE: 0.09); for the 45–64 age group, the ARIMA model (RMSE: 0.46) outperformed the Prophet model (RMSE: 0.87); and for the ≥65 age group, the Prophet model (RMSE: 3.25) outperformed the ARIMA model (RMSE: 3.83) (Figure 4).

### Urban–rural disparities in mortality (1999–2024; projections to 2035)

**Figure 5:**
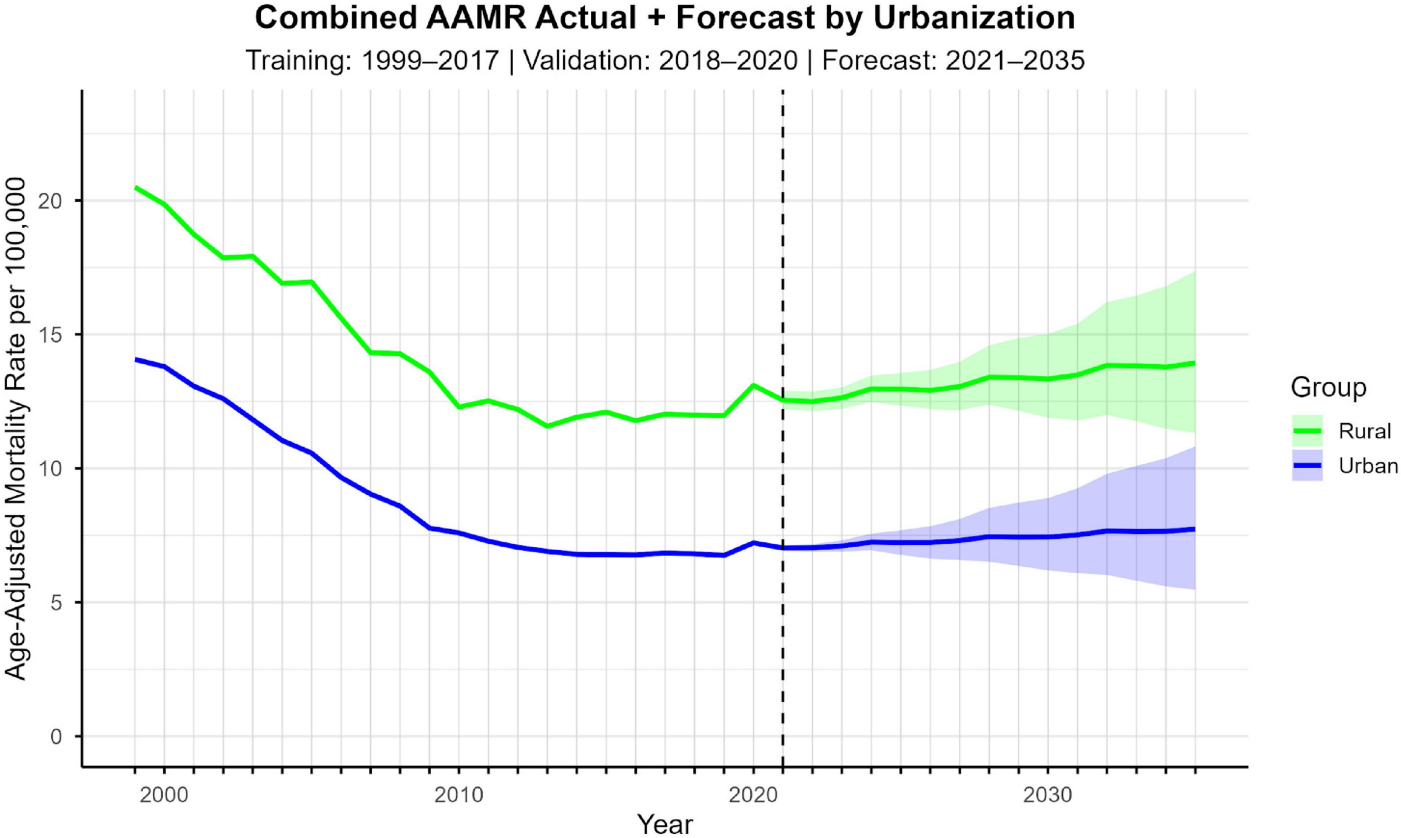
Age-adjusted mortality rates for MI- and HF-related deaths among adults aged ≥25 years, stratified by urbanization level, per 100,000 population, with projections to 2035.

Clear urban–rural differences in mortality were observed. The mean AAMR was 14.55 per 100,000 in rural populations compared with 9.04 per 100,000 in urban populations (Supplementary Table 7). Rural mortality remained consistently higher than urban mortality throughout the study period. Urban mortality declined significantly until 2014, followed by a modest increase through 2020, whereas rural mortality declined until 2013, followed by a mild increase thereafter (Supplementary Table 2). By 2035, projected AAMRs are 13.94 per 100,000 (95% CI: 11.18–17.29) in rural populations and 7.73 per 100,000 (95% CI: 5.60–10.96) in urban populations (Supplementary Table 12). For rural populations, the Prophet model (RMSE: 0.58) outperformed the ARIMA model (RMSE: 0.87). For urban populations, the Prophet model (RMSE: 0.25) outperformed the ARIMA model (RMSE: 0.30) (Figure 5).

### Sensitivity analysis

**Figure 6:**
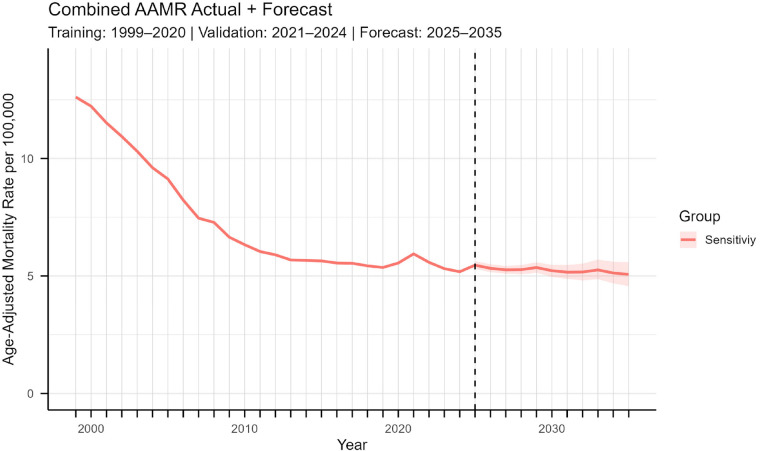
Sensitivity analysis of age-adjusted mortality rates for heart failure-related deaths among patients with myocardial infarction, where heart failure was treated as a multiple cause of death, with projections to 2035.

Sensitivity analyses evaluating HF as a multiple cause of death with MI as the underlying cause demonstrated findings consistent with the primary analysis. Projected mortality rates gradually declined between 2025 and 2035, with the AAMR decreasing from 5.46 (95% CI: 5.29–5.62) in 2025 to 5.06 (95% CI: 4.57–5.63) in 2035 (Supplementary Table 13). The Prophet model (RMSE: 0.36) outperformed the ARIMA model (RMSE: 0.38) (Figure 6).

## Discussion

In this 25-year analysis of mortality data obtained from the Centers for Disease Control and Prevention, several key findings were associated with myocardial infarction (MI) and heart failure (HF), depicting 552,835 deaths nationwide. Stratification up to 2035 showed a high death ratio. Noticeable disparities in the mortality trend were observed when stratified by sex, race, census region, age, and urbanization. Males consistently showed a higher age-adjusted mortality rate (AAMR) throughout the study period, while females demonstrated a greater decline. Moreover, a decline in AAMR was observed when the findings were stratified by age and census region. Non-Hispanic (NH) Blacks exhibited the greatest AAMR, with concerning future projections in mortality rates. Although age-stratified analyses demonstrated declining AAMRs within several age groups, the projected increase in overall mortality may reflect population aging, growth in cardiometabolic risk factors, and disproportionate burden among vulnerable subpopulations. However, the rural population showed a rise in mortality rates, with future predictions placing them above the urban population. These findings highlight the need for targeted strategies that address vulnerable populations, providing swift treatment and preventing further complications (Figure 6).

**Figure 7:**
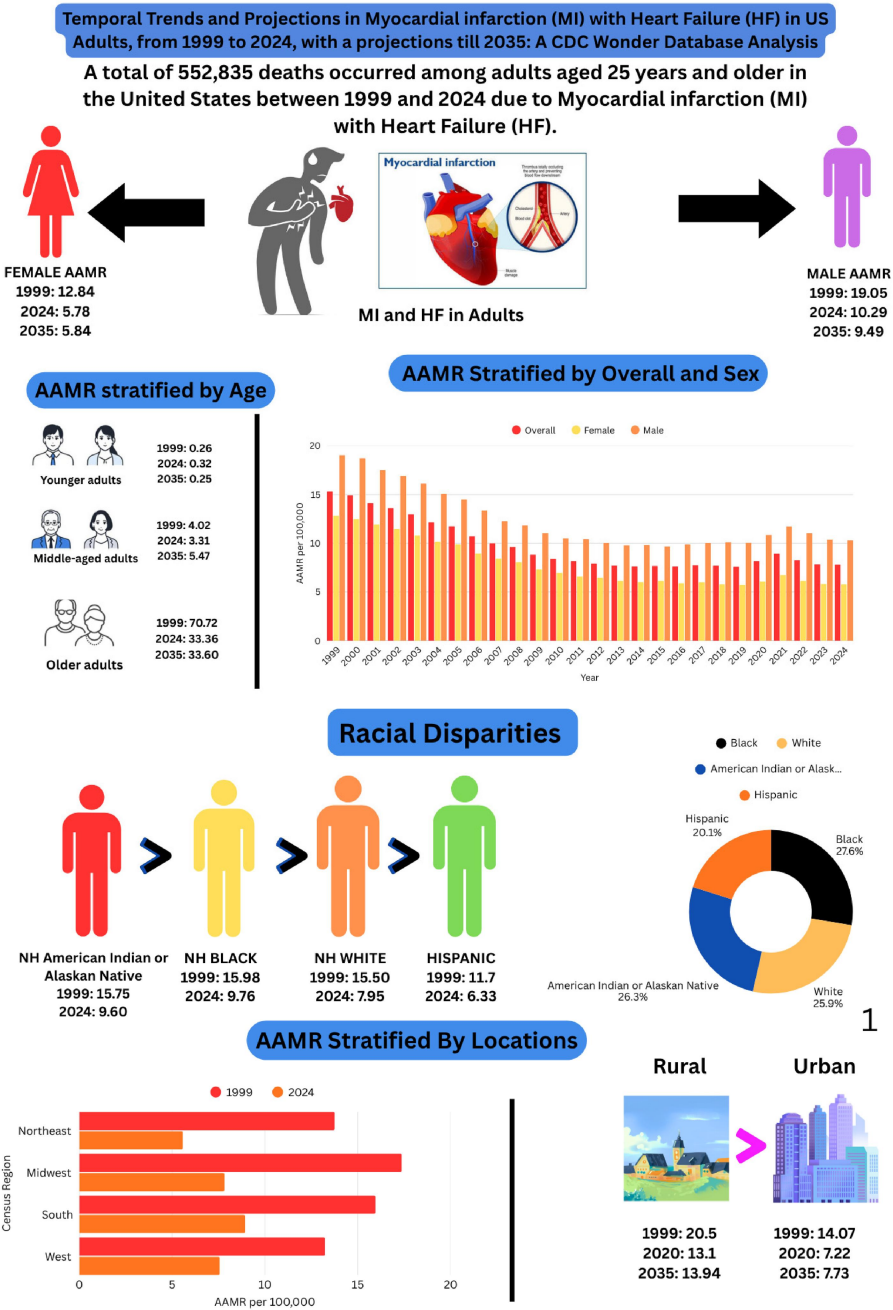
Central Illustration. Trends and projections of MI- and HF-related mortality among U.S. adults aged ≥25 years, 1999–2035.

Annual trends revealed a 1.96-fold decline in AAMR (AAMR declining from 15.32 in 1999 to 7.81 in 2024). Several factors can be attributed to this decline, including population-wide and clinical strategies to reduce risk factors for MI and HF—such as blood pressure, serum cholesterol, smoking, obesity, diabetes, and physical inactivity. The benefits were largely seen in low- and moderate-risk individuals rather than those at high risk (24). Moreover, implementing strategies involving joint risk-factor control, particularly for hypertension, has proven effective in lowering the incidence of MI and consequently HF (25). Regulating blood pressure prevents volume overload, while reducing smoking prevents “arterial stiffness, oxidative stress, and inflammation” (25). However, this decline continued only until 2012, after which a significant rise in mortality was observed. This resurgence is largely attributed to disparities in healthcare access, inadequately controlled modifiable risk factors—such as obesity, impacting nearly 208 million people in the U.S.—and metabolic risks (8, 26). The projected 10% rise in AAMR by 2035 may be linked to future increases in obesity and other risk factors, particularly among younger populations. This underscores the urgent need for government programs to raise awareness early and mitigate these forecasted trends (26).

Males consistently show higher AAMR, reflecting disparities in mortality trends and a greater lifetime risk of cardiovascular disease—38% in males and 24% in females (27). This may be due to a higher prevalence of smoking, hypertension (50.8% in males vs. 44.6% in females), and diabetes in men, increasing susceptibility to coronary artery blockage and consequent HF (28–31). This is further supported by a 2.2-fold decrease in AAMR in females (AAMR falling from 12.84 in 1999 to 5.78 in 2024) compared with a 1.85-fold decrease in males (AAMR decreasing from 19.05 in 1999 to 10.29 in 2024). Lifestyle modifications such as hypertension control, hyperlipidemia management, smoking cessation, increased statin use, awareness campaigns, and greater focus on women's cardiovascular health could play significant roles in curbing the rising incidence among females (32). With projections continuing to place males above females in terms of AAMR, there is a need for equitable preventive strategies involving early lifestyle interventions and gender-specific approaches, along with access to prompt treatment.

All racial groups demonstrated declines in mortality rates, with the greatest decrease observed among NH White individuals (1.95-fold), followed by Hispanic individuals (1.85-fold), American Indian, and NH Black populations. NH Blacks consistently showed higher AAMR compared to other groups, with a significant rise after 2013, highlighting a greater burden of risk factors and comorbidities, including hypertension, obesity, and diabetes (33, 34). Additionally, lower income, education, and socioeconomic disadvantages correlate with poorer cardiovascular health and higher incidence rates among NH Black Americans, particularly in younger populations (35). A recent article suggested NH Blacks have a 3.8% lower Mean Life's Essential 8 score with 89% higher incidence of low Cardiovascular health compared to NH White especially in states subjected to structural racism (36). Nonetheless, the overall decline in AAMR across racial groups may reflect the success of government-funded programs such as Million Hearts and the National Hypertension Control Program, which focus on risk-factor control and optimized care (37, 38). Although mortality rates declined across all racial groups, the decline was slower among NH Black individuals, resulting in persistent and in some projections widening relative disparities in AAMR compared with other racial groups. Future projections estimated NH Blacks to have a 71% higher AAMR compared to Hispanics, 22.5% higher than NH White individuals and 19.0% higher than NH American Indian individuals. With projections placing NH Blacks among the groups with the highest AAMRs, there is an urgent need to expand these programs to disadvantaged individuals and ensure equitable treatment access to reduce future disparities (Figure 7).

Analysis of census regions revealed geographic variation in MI and HF mortality across the United States. The Northeast experienced the steepest decline in mortality, followed by the Midwest and West, whereas the South consistently demonstrated the highest mortality rates between 1999 and 2024. Despite national progress in cardiovascular prevention—including declines in smoking prevalence (39), broader cholesterol screening and lipid control (40, 41), and improved acute and chronic cardiovascular care (42, 43)—the South remains disproportionately affected. This likely reflects concentrations of cardiometabolic risk factors, limited access to specialized cardiac care, and lower socioeconomic status. Projections indicate that by 2035, the South will continue to lead in MI- and HF-related deaths, while the Northeast will maintain the lowest rates.

Age-specific analyses revealed diverging trends across the study period. Older adults (≥65 years) exhibited significant and sustained mortality declines, reflecting improvements in secondary prevention, early revascularization, and HF management (43). However, among younger adults (25–44 years), mortality began to rise noticeably after 2010 and continues to increase. This troubling trend may be linked to higher rates of obesity, diabetes, and substance use—particularly smoking—as well as diagnostic delays in this group (44). Middle-aged adults (45–64 years) initially showed mortality reductions until 2012, followed by a mild rebound, suggesting prior cardiovascular health gains. These findings align with the CDC *Heart Disease and Stroke Statistics 2025* report, which notes stagnation in overall cardiovascular mortality primarily driven by worsening trends in younger and middle-aged populations (45).

Urban–rural analyses revealed clear differences in mortality trends. Urban areas experienced a steady decline, while rural populations showed an initial decrease followed by a rebound after 2013. By 2035, forecasted mortality in rural areas is expected to remain nearly twice that of urban populations. This growing disparity indicates persistent inequities in healthcare infrastructure, emergency response times, and access to preventive and specialized cardiac care (46).

These regional trends are consistent with recent CDC and AHA surveillance data identifying a “Heart Disease Belt” across southern states, emphasizing the need for regionally tailored interventions focusing on hypertension control, obesity prevention, and health equity (47). The age-specific analyses also highlight the need for early prevention strategies targeting lifestyle and behavioral risk factors among younger adults, alongside improved early detection. Public health efforts should aim to reverse the urban–rural disparity through the expansion of telecardiology networks, rural hospital support, and equitable access to evidence-based therapies (48).

### Limitations

This study is subject to several limitations. First, it relies on CDC WONDER data obtained from death certificates, which may be susceptible to misclassification—particularly in differentiating between primary and contributory causes of death. Second, changes in diagnostic criteria and coding practices over the study period may have influenced observed mortality trends. Third, the analysis of comorbidities was restricted to a limited set of conditions and therefore did not capture the full spectrum of coexisting diseases. Fourth, this study did not evaluate treatment adherence, socioeconomic status, or lifestyle factors that may affect outcomes. Fifth, the time-series forecasting techniques employed—ARIMA and Prophet—carry inherent methodological constraints. ARIMA assumes linearity and stationarity, limiting its ability to model nonlinear dynamics or long-term fluctuations, whereas Prophet may oversimplify complex temporal relationships and is sensitive to changepoint selection, seasonal assumptions, and parameter configurations. Furthermore, urbanization data were available only through 2020, restricting the forecasting horizon and potentially limiting the accuracy of post-2020 projections. Despite these limitations, the use of a large, nationally representative dataset offers a comprehensive overview of mortality trends and disparities, yielding valuable insights for researchers, clinicians, and policymakers.

## Conclusion

From 1999 to 2024, mortality due to MI and HF declined significantly across the United States, with AAMR decreasing from 15.32 to 7.81 per 100,000 population. The decline was most pronounced in the early years (1999–2012), followed by periods of stabilization or modest increases in certain subgroups. Men, non-Hispanic Black individuals, older adults, residents in the South and Midwest, and rural populations consistently exhibited higher mortality rates compared with women, Hispanic individuals, younger adults, residents in the Northeast, and urban populations. Despite overall progress, disparities persist by sex, race, age, region, and urbanization. Projections to 2035 suggest continued reductions in most groups, though rural residents, older adults, and non-Hispanic Black individuals are expected to remain at higher risk. These findings underscore the need for targeted interventions to address persistent disparities and sustain declines in cardiovascular mortality.

## Data Availability

Publicly available datasets were analyzed in this study. This data can be found here: https://wonder.cdc.gov.
