# Supplementary material for: Temporal trends and projected mortality of myocardial infarction and heart failure in the United States, 1999–2035: a CDC WONDER analysis
Source: Front Cardiovasc Med. 2026 Jul 9;13:1772415. doi: 10.3389/fcvm.2026.1772415 (PMC13391839; doi:10.3389/fcvm.2026.1772415)
Supplement: Supplementary file 1 [file Datasheet1.docx]

**Supplemental Table 1, Heart Failure-related mortalities in Patients with Myocardial Infarction, Stratified by Sex and Race, in the United States, 1999 to 2024**

| **Supplemental Table 1, Heart Failure-related mortalities in Patients with Myocardial Infarction, Stratified by Sex and Race, in the United States, 1999 to 2024** | | | | | | | | |
| --- | --- | --- | --- | --- | --- | --- | --- | --- |
| **Deaths** | | | | | | | | |
| **Year** | **Overall** | **Women** | **Men** | **NH American Indian or Alaska Native** | **NH Whites** | **Hispanic** | **NH Blacks** | **Population** |
| **1999** | 26905 | 14323 | 12582 | 101 | 23247 | 891 | 2289 | 180408769 |
| **2000** | 26654 | 14134 | 12520 | 84 | 23019 | 922 | 2281 | 181984640 |
| **2001** | 25598 | 13648 | 11950 | 66 | 22081 | 968 | 2132 | 184305128 |
| **2002** | 25001 | 13239 | 11762 | 103 | 21297 | 966 | 2191 | 186208028 |
| **2003** | 24227 | 12750 | 11477 | 114 | 20790 | 971 | 2000 | 188090429 |
| **2004** | 23022 | 12066 | 10956 | 103 | 19656 | 934 | 1971 | 190205384 |
| **2005** | 22748 | 11942 | 10806 | 63 | 19208 | 977 | 2091 | 192551384 |
| **2006** | 21263 | 11006 | 10257 | 93 | 17865 | 978 | 1899 | 195019359 |
| **2007** | 20232 | 10527 | 9705 | 85 | 16920 | 973 | 1866 | 197403777 |
| **2008** | 19885 | 10293 | 9592 | 81 | 16704 | 963 | 1770 | 199795090 |
| **2009** | 18655 | 9460 | 9195 | 99 | 15597 | 911 | 1660 | 202107016 |
| **2010** | 18126 | 9150 | 8976 | 73 | 15116 | 885 | 1663 | 203891983 |
| **2011** | 18126 | 8922 | 9204 | 102 | 15060 | 931 | 1665 | 206592936 |
| **2012** | 18012 | 8869 | 9143 | 105 | 14908 | 946 | 1645 | 208826037 |
| **2013** | 17894 | 8624 | 9270 | 98 | 14647 | 965 | 1732 | 211085314 |
| **2014** | 18206 | 8611 | 9595 | 111 | 14768 | 1065 | 1794 | 213809280 |
| **2015** | 18674 | 8913 | 9761 | 101 | 15009 | 1146 | 1897 | 216553817 |
| **2016** | 18941 | 8712 | 10229 | 127 | 15158 | 1212 | 1931 | 218641417 |
| **2017** | 19622 | 9000 | 10622 | 119 | 15528 | 1322 | 2049 | 221447331 |
| **2018** | 19905 | 8811 | 11094 | 134 | 15763 | 1377 | 2017 | 223311190 |
| **2019** | 20111 | 8820 | 11291 | 154 | 15703 | 1503 | 2198 | 224981167 |
| **2020** | 21994 | 9479 | 12515 | 181 | 16798 | 1802 | 2538 | 226635013 |
| **2021** | 23354 | 10069 | 13285 | 176 | 17852 | 1855 | 2658 | 228238412 |
| **2022** | 22706 | 9744 | 12962 | 151 | 17447 | 1756 | 2550 | 229508599 |
| **2023** | 21568 | 9123 | 12445 | 122 | 16373 | 1724 | 2607 | 231529762 |
| **2024** | 21406 | 9043 | 12363 | 174 | 17778 | 1719 | 2639 | 235615087 |
| **Total** | 552835 | 269278 | 283557 | 2920 | 454,292 | 30,662 | 53733 | 5,398,746,349 |

**Supplemental Table 2, Annual percent change (APC) of Heart Failure-related Age-Adjusted Mortality Rates per 100,000, in Patients with Myocardial Infarction in the United States, 1999 to 2024**

| **Supplemental Table 2, Annual percent change (APC) of Heart Failure-related Age-Adjusted Mortality Rates per 100,000, in Patients with Myocardial Infarction in the United States, 1999 to 2024** | | |
| --- | --- | --- |
| **Year Interval** | **APC (95% CI)** | **P-value** |
| **Overall** | | |
| 1999–2012 | -5.39* (-5.98 to -4.89) | < 0.000001 |
| 2012–2024 | 0.35 (-0.32 to 1.18) | 0.259548 |
| **Men (Male)** | | |
| 1999–2012 | -5.27* (-6.00 to -4.67) | < 0.000001 |
| 2012–2024 | 0.94* (0.22 to 1.84) | 0.012398 |
| **Women (Female)** | | |
| 1999–2013 | -5.49* (-6.03 to -5.03) | < 0.000001 |
| 2013–2024 | -0.11 (-0.90 to 0.86) | 0.783443 |
| **NH White** | | |
| 1999–2012 | -5.32* (-5.90 to -4.83) | < 0.000001 |
| 2012–2024 | 0.36 (-0.32 to 1.17) | 0.261148 |
| **NH Black or African American** | | |
| 1999–2012 | -5.30* (-6.10 to -4.59) | < 0.000001 |
| 2012–2024 | 1.44* (0.65 to 2.48) | 0.0004 |
| **NH American Indian or Alaska Native** | | |
| 1999–2024 | -1.71* (-2.76 to -0.44) | 0.010398 |
| **Hispanic** | | |
| 1999–2014 | -5.28* (-5.94 to -4.71) | < 0.000001 |
| 2014–2020 | 4.52* (2.96 to 6.92) | 0.003199 |
| 2020–2024 | -3.84* (-6.08 to -1.37) | 0.003199 |
| **Northeast region** | | |
| 1999–2004 | -4.31* (-5.93 to -2.52) | < 0.000001 |
| 2004–2009 | -7.60* (-8.47 to -1.18) | 0.004799 |
| 2009–2024 | -2.00* (-2.56 to -1.36) | 0.007598 |
| **Midwest region** | | |
| 1999–2011 | -6.36* (-7.07 to -5.74) | < 0.000001 |
| 2011–2024 | 0.32 (-0.34 to 1.09) | 0.304339 |
| **South region** | | |
| 1999–2013 | -5.12* (-5.71 to -4.61) | < 0.000001 |
| 2013–2024 | 1.23* (0.45 to 2.17) | 0.004399 |
| **West region** | | |
| 1999–2012 | -4.65* (-5.47 to -4.00) | < 0.000001 |
| 2012–2024 | 0.61 (-0.16 to 1.67) | 0.113577 |
| **Younger Adults (25-44)** | | |
| 1999–2005 | 2.81 (-4.04 to 13.39) | 0.240352 |
| 2005–2010 | -11.24 (-15.18 to 9.22) | 0.116777 |
| 2010–2024 | 6.31* (3.38 to 10.06) | 0.023995 |
| **Middle Aged Adults (45-64)** | | |
| 1999–2012 | -4.34* (-5.09 to -3.68) | < 0.000001 |
| 2012–2020 | 5.30* (4.03 to 7.99) | 0.031994 |
| 2020–2024 | -0.23 (-2.33 to 2.60) | 0.986603 |
| **Older Adults (65+)** | | |
| 1999–2012 | -5.56* (-6.13 to -5.08) | < 0.000001 |
| 2012–2024 | -0.20 (-0.83 to 0.55) | 0.544691 |
| **Rural areas** | | |
| 1999-2013 | -4.08* (-4.49 to -3.72) | <0.000001 |
| 2013-2020 | 1.21* (0.14 to 2.66) | 0.032793 |
| **Urban areas** | | |
| 1999-2002 | -3.71* (-4.88 to -1.83) | <0.000001 |
| 2002-2010 | -6.37* (-7.22 to -6.04) | <0.000001 |
| 2010-2014 | -2.63* (-4.65 to -1.05) | 0.0008 |
| 2014-2020 | 0.70* (0.10 to 1.76) | 0.023195 |
| APC = annual percent change; NH = non-Hispanic; * indicates that the annual percentage change (APC) is significantly different from zero at α = 0.05. AAMR = age-adjusted mortality rate. The data for urbanization is only available till 2020 in the CDC Wonder Database. | | |

**Supplemental Table 3, Overall and Sex‐Stratified Heart Failure-Related Age-Adjusted Mortality Rates per 100,000, in Patients with Myocardial Infarction in the United States, 1999 to 2024**

| **Supplemental Table 3, Overall and Sex‐Stratified Heart Failure-Related Age-Adjusted Mortality Rates per 100,000, in Patients with Myocardial Infarction in the United States, 1999 to 2024** | | | |
| --- | --- | --- | --- |
| **Year** | **Men AAMR (95% CI)** | **Women AAMR (95% CI)** | **Overall AAMR (95% CI)** |
| **1999** | 19.05 (18.71–19.39) | 12.84 (12.63–13.06) | 15.32 (15.14–15.51) |
| **2000** | 18.73 (18.40–19.07) | 12.49 (12.29–12.70) | 14.94 (14.76–15.12) |
| **2001** | 17.52 (17.20–17.84) | 11.93 (11.72–12.13) | 14.11 (13.94–14.29) |
| **2002** | 16.93 (16.62–17.25) | 11.44 (11.24–11.64) | 13.62 (13.45–13.79) |
| **2003** | 16.13 (15.83–16.43) | 10.82 (10.63–11.01) | 12.98 (12.81–13.14) |
| **2004** | 15.06 (14.77–15.34) | 10.15 (9.97–10.34) | 12.16 (12.00–12.31) |
| **2005** | 14.50 (14.22–14.77) | 9.88 (9.70–10.06) | 11.75 (11.60–11.91) |
| **2006** | 13.35 (13.09–13.61) | 8.95 (8.78–9.12) | 10.73 (10.58–10.87) |
| **2007** | 12.26 (12.01–12.51) | 8.42 (8.26–8.58) | 10.00 (9.86–10.13) |
| **2008** | 11.84 (11.60–12.09) | 8.07 (7.91–8.23) | 9.63 (9.50–9.77) |
| **2009** | 11.03 (10.80–11.26) | 7.34 (7.19–7.49) | 8.84 (8.71–8.96) |
| **2010** | 10.53 (10.31–10.75) | 6.97 (6.82–7.11) | 8.41 (8.28–8.53) |
| **2011** | 10.42 (10.20–10.63) | 6.59 (6.45–6.73) | 8.17 (8.05–8.29) |
| **2012** | 10.01 (9.80–10.22) | 6.46 (6.32–6.59) | 7.94 (7.82–8.06) |
| **2013** | 9.80 (9.60–10.00) | 6.15 (6.01–6.28) | 7.71 (7.59–7.82) |
| **2014** | 9.83 (9.63–10.03) | 6.02 (5.89–6.15) | 7.64 (7.53–7.75) |
| **2015** | 9.69 (9.50–9.89) | 6.15 (6.02–6.28) | 7.67 (7.56–7.79) |
| **2016** | 9.87 (9.68–10.07) | 5.91 (5.79–6.04) | 7.62 (7.51–7.73) |
| **2017** | 10.01 (9.81–10.20) | 6.01 (5.88–6.13) | 7.74 (7.63–7.85) |
| **2018** | 10.11 (9.92–10.30) | 5.79 (5.67–5.91) | 7.69 (7.58–7.80) |
| **2019** | 10.05 (9.86–10.24) | 5.74 (5.62–5.86) | 7.60 (7.49–7.71) |
| **2020** | 10.86 (10.67–11.06) | 6.09 (5.96–6.21) | 8.17 (8.07–8.28) |
| **2021** | 11.73 (11.52–11.93) | 6.75 (6.62–6.89) | 8.92 (8.81–9.04) |
| **2022** | 11.04 (10.85–11.23) | 6.16 (6.03–6.28) | 8.26 (8.15–8.36) |
| **2023** | 10.38 (10.20–10.57) | 5.83 (5.71–5.95) | 7.84 (7.73–7.94) |
| **2024** | 9.92 (9.74–10.10) | 5.61 (5.49–5.73) | 7.55 (7.45–7.65) |
| **Total (1999–2024)** | 12.33 (11.90–12.76) | 7.87 (7.52–8.22) | 9.73 (9.39–10.07) |

*“Total” represents the mean age-adjusted mortality rate per 100,000 population.*

**Supplemental Table 4, Race‐Stratified Heart Failure-Related Age-Adjusted Mortality Rates per 100,000 in Patients with Myocardial Infarction in the United States, 1999 to 2024**

| **Spplemental Table 4, Race‐Stratified Heart Failure-Related Age-Adjusted Mortality Rates per 100,000 in Patients with Myocardial Infarction in the United States, 1999 to 2024** | | | | |
| --- | --- | --- | --- | --- |
| **Year** | **Hispanic (95% CI)** | **NH American Indian or Alaska Native (95% CI)** | **NH Black (95% CI)** | **NH White (95% CI)** |
| **1999** | 11.70 (10.90–12.49) | 15.75 (12.53–18.97) | 15.98 (15.32–16.64) | 15.50 (15.30–15.70) |
| **2000** | 11.47 (10.70–12.23) | 12.36 (9.77–15.43) | 15.66 (15.01–16.31) | 15.19 (14.99–15.39) |
| **2001** | 11.23 (10.50–11.96) | 9.35 (7.14–12.04) | 14.39 (13.77–15.01) | 14.37 (14.18–14.56) |
| **2002** | 10.65 (9.96–11.35) | 12.95 (10.30–15.60) | 14.54 (13.92–15.15) | 13.71 (13.53–13.89) |
| **2003** | 10.26 (9.59–10.93) | 14.84 (11.98–17.70) | 13.14 (12.55–13.72) | 13.21 (13.03–13.38) |
| **2004** | 9.41 (8.79–10.03) | 12.98 (10.32–15.63) | 12.71 (12.14–13.28) | 12.35 (12.18–12.52) |
| **2005** | 9.27 (8.67–9.87) | 7.77 (5.89–10.07) | 13.02 (12.45–13.59) | 11.84 (11.68–12.01) |
| **2006** | 8.78 (8.21–9.35) | 11.19 (8.91–13.87) | 11.52 (10.99–12.04) | 10.86 (10.70–11.02) |
| **2007** | 8.16 (7.63–8.69) | 8.95 (7.04–11.22) | 10.95 (10.44–11.46) | 10.11 (9.96–10.27) |
| **2008** | 7.73 (7.22–8.23) | 8.95 (7.00–11.27) | 10.07 (9.59–10.55) | 9.80 (9.65–9.95) |
| **2009** | 6.73 (6.28–7.19) | 11.19 (8.99–13.77) | 9.27 (8.82–9.73) | 9.03 (8.89–9.17) |
| **2010** | 6.40 (5.97–6.84) | 7.73 (5.98–9.84) | 9.05 (8.61–9.50) | 8.59 (8.46–8.73) |
| **2011** | 6.28 (5.86–6.69) | 9.42 (7.49–11.35) | 8.67 (8.24–9.10) | 8.39 (8.26–8.53) |
| **2012** | 5.91 (5.53–6.30) | 9.72 (7.78–11.67) | 8.30 (7.89–8.71) | 8.18 (8.04–8.31) |
| **2013** | 5.74 (5.37–6.11) | 8.00 (6.42–9.86) | 8.33 (7.92–8.73) | 7.90 (7.77–8.03) |
| **2014** | 5.89 (5.53–6.26) | 9.03 (7.27–10.79) | 8.29 (7.90–8.69) | 7.84 (7.71–7.97) |
| **2015** | 5.98 (5.62–6.33) | 8.14 (6.49–9.79) | 8.45 (8.06–8.84) | 7.84 (7.71–7.96) |
| **2016** | 5.97 (5.62–6.31) | 9.45 (7.74–11.15) | 8.38 (8.00–8.76) | 7.80 (7.68–7.93) |
| **2017** | 6.15 (5.81–6.49) | 8.20 (6.68–9.73) | 8.50 (8.12–8.88) | 7.89 (7.76–8.01) |
| **2018** | 6.07 (5.74–6.39) | 8.49 (7.01–9.98) | 8.15 (7.78–8.51) | 7.88 (7.75–8.00) |
| **2019** | 6.39 (6.06–6.72) | 9.65 (8.08–11.22) | 8.76 (8.39–9.14) | 7.75 (7.63–7.87) |
| **2020** | 7.27 (6.92–7.61) | 10.71 (9.11–12.32) | 9.75 (9.36–10.14) | 8.21 (8.08–8.33) |
| **2021** | 7.39 (7.04–7.73) | 10.92 (9.25–12.59) | 10.31 (9.90–10.71) | 9.19 (9.05–9.32) |
| **2022** | 6.69 (6.37–7.02) | 9.31 (7.79–10.82) | 9.67 (9.28–10.06) | 8.56 (8.43–8.69) |
| **2023** | 6.36 (6.05–6.67) | 7.17 (5.88–8.47) | 9.68 (9.30–10.06) | 8.05 (7.93–8.18) |
| **2024** | 5.91 (5.63–6.21) | 6.84 (5.84–7.97) | 8.98 (8.64–9.34) | 7.58 (7.46–7.69) |
| **Total (1999–2024)** | 7.70 (7.52–7.88) | 10.07 (9.49–10.65) | 10.59 (10.40–10.78) | 9.92 (9.78–10.06) |

**Supplemental Table 5, Heart Failure-related Age-Adjusted Mortality Rates per 100,000, Stratified by Census Region, in Patients with Myocardial Infarction in the United States, 1999 to 2024**

| **Supplemental Table 5, Heart Failure-related Age-Adjusted Mortality Rates per 100,000, Stratified by Census Region, in Patients with Myocardial Infarction in the United States, 1999 to 2024** | | | | |
| --- | --- | --- | --- | --- |
| **Year** | **Northeast (95% CI)** | **Midwest (95% CI)** | **South (95% CI)** | **West (95% CI)** |
| **1999** | 13.76 (13.39–14.14) | 17.37 (16.97–17.77) | 15.95 (15.63–16.26) | 13.24 (12.86–13.63) |
| **2000** | 13.47 (13.10–13.84) | 16.24 (15.86–16.62) | 16.14 (15.83–16.46) | 12.82 (12.45–13.20) |
| **2001** | 13.11 (12.75–13.47) | 15.43 (15.06–15.80) | 14.96 (14.66–15.26) | 12.10 (11.74–12.46) |
| **2002** | 12.29 (11.95–12.64) | 14.62 (14.27–14.98) | 14.48 (14.18–14.77) | 12.17 (11.82–12.53) |
| **2003** | 11.47 (11.13–11.80) | 13.87 (13.53–14.22) | 13.76 (13.47–14.05) | 11.95 (11.60–12.30) |
| **2004** | 10.96 (10.63–11.28) | 12.49 (12.16–12.82) | 13.00 (12.73–13.28) | 11.32 (10.98–11.66) |
| **2005** | 10.74 (10.42–11.06) | 12.16 (11.84–12.48) | 12.62 (12.35–12.89) | 10.63 (10.31–10.95) |
| **2006** | 9.51 (9.21–9.81) | 11.24 (10.94–11.55) | 11.55 (11.29–11.80) | 9.93 (9.62–10.24) |
| **2007** | 8.84 (8.56–9.13) | 9.93 (9.65–10.22) | 11.03 (10.79–11.28) | 9.29 (9.00–9.58) |
| **2008** | 8.23 (7.96–8.50) | 9.77 (9.49–10.05) | 10.57 (10.34–10.81) | 9.03 (8.75–9.32) |
| **2009** | 7.52 (7.26–7.77) | 9.04 (8.77–9.30) | 9.66 (9.44–9.88) | 8.38 (8.11–8.65) |
| **2010** | 7.60 (7.34–7.86) | 8.35 (8.09–8.60) | 9.19 (8.98–9.41) | 7.95 (7.69–8.21) |
| **2011** | 7.32 (7.07–7.58) | 8.32 (8.06–8.57) | 8.66 (8.45–8.86) | 8.01 (7.75–8.26) |
| **2012** | 7.05 (6.80–7.30) | 8.13 (7.89–8.38) | 8.57 (8.36–8.77) | 7.37 (7.12–7.61) |
| **2013** | 6.99 (6.75–7.23) | 7.93 (7.69–8.18) | 8.13 (7.93–8.32) | 7.20 (6.96–7.44) |
| **2014** | 6.69 (6.45–6.93) | 8.00 (7.76–8.24) | 8.22 (8.02–8.41) | 7.11 (6.88–7.34) |
| **2015** | 6.65 (6.42–6.89) | 7.88 (7.64–8.12) | 8.16 (7.97–8.35) | 7.48 (7.24–7.71) |
| **2016** | 6.52 (6.29–6.75) | 7.75 (7.52–7.99) | 8.05 (7.86–8.23) | 7.61 (7.38–7.84) |
| **2017** | 6.22 (6.00–6.45) | 8.10 (7.86–8.34) | 8.23 (8.05–8.42) | 7.68 (7.45–7.91) |
| **2018** | 6.17 (5.95–6.39) | 8.02 (7.79–8.26) | 8.32 (8.13–8.50) | 7.49 (7.26–7.71) |
| **2019** | 5.84 (5.63–6.06) | 7.85 (7.62–8.09) | 8.32 (8.13–8.50) | 7.56 (7.34–7.79) |
| **2020** | 6.26 (6.04–6.48) | 8.53 (8.29–8.77) | 8.98 (8.80–9.17) | 7.90 (7.68–8.13) |
| **2021** | 6.42 (6.20–6.65) | 9.35 (9.10–9.61) | 9.93 (9.73–10.13) | 8.92 (8.67–9.17) |
| **2022** | 6.02 (5.80–6.23) | 8.65 (8.40–8.89) | 9.13 (8.94–9.31) | 8.31 (8.08–8.54) |
| **2023** | 5.64 (5.44–5.85) | 8.11 (7.88–8.35) | 8.85 (8.67–9.04) | 7.59 (7.37–7.81) |
| **2024** | 5.40 (5.20–5.61) | 7.60 (7.38–7.83) | 8.64 (8.47–8.82) | 7.29 (7.08–7.50) |
| **Total (1999–2024)** | 8.34 (8.18–8.50) | 10.19 (10.01–10.37) | 10.52 (10.37–10.67) | 9.10 (8.92–9.28) |

*“Total” represents the mean age-adjusted mortality rate per 100,000 population.*

**Supplemental Table 6, Age‐Stratified Heart Failure-Related Age-Adjusted Mortality Rates per 100,000, in Patients with Myocardial Infarction in the United States, 1999 to 2024**

| **Supplemental Table 6, Age‐Stratified Heart Failure-Related Age-Adjusted Mortality Rates per 100,000, in Patients with Myocardial Infarction in the United States, 1999 to 2024** | | | |
| --- | --- | --- | --- |
| **Year** | **Younger Adults (25–44) (95% CI)** | **Middle-Aged Adults (45–64) (95% CI)** | **Older Adults (65+) (95% CI)** |
| **1999** | 0.23 (0.19–0.26) | 4.00 (3.84–4.16) | 69.83 (68.95–70.70) |
| **2000** | 0.21 (0.18–0.24) | 4.14 (3.98–4.30) | 68.34 (67.48–69.21) |
| **2001** | 0.22 (0.18–0.25) | 3.61 (3.47–3.76) | 65.42 (64.57–66.26) |
| **2002** | 0.25 (0.22–0.28) | 3.56 (3.42–3.71) | 63.10 (62.28–63.93) |
| **2003** | 0.21 (0.18–0.24) | 3.41 (3.28–3.55) | 60.50 (59.70–61.31) |
| **2004** | 0.23 (0.20–0.27) | 3.28 (3.15–3.42) | 56.63 (55.85–57.40) |
| **2005** | 0.21 (0.18–0.25) | 3.27 (3.13–3.40) | 55.07 (54.31–55.83) |
| **2006** | 0.21 (0.18–0.24) | 3.07 (2.94–3.20) | 50.54 (49.82–51.26) |
| **2007** | 0.24 (0.21–0.28) | 2.96 (2.84–3.08) | 46.93 (46.24–47.62) |
| **2008** | 0.18 (0.15–0.21) | 2.81 (2.70–2.93) | 45.19 (44.52–45.86) |
| **2009** | 0.16 (0.14–0.19) | 2.70 (2.58–2.81) | 41.28 (40.65–41.91) |
| **2010** | 0.15 (0.12–0.17) | 2.65 (2.54–2.76) | 39.36 (38.74–39.97) |
| **2011** | 0.19 (0.16–0.22) | 2.58 (2.47–2.69) | 38.26 (37.66–38.85) |
| **2012** | 0.16 (0.13–0.19) | 2.58 (2.47–2.69) | 36.47 (35.90–37.04) |
| **2013** | 0.17 (0.15–0.20) | 2.65 (2.54–2.76) | 34.77 (34.22–35.32) |
| **2014** | 0.18 (0.15–0.21) | 2.87 (2.76–2.99) | 33.85 (33.32–34.38) |
| **2015** | 0.19 (0.16–0.22) | 2.95 (2.83–3.06) | 33.58 (33.06–34.10) |
| **2016** | 0.17 (0.14–0.20) | 3.01 (2.89–3.12) | 33.03 (32.52–33.54) |
| **2017** | 0.22 (0.19–0.25) | 3.17 (3.05–3.29) | 32.95 (32.45–33.45) |
| **2018** | 0.23 (0.19–0.26) | 3.30 (3.17–3.42) | 32.31 (31.83–32.80) |
| **2019** | 0.24 (0.21–0.28) | 3.32 (3.19–3.44) | 31.69 (31.22–32.17) |
| **2020** | 0.29 (0.25–0.32) | 3.97 (3.83–4.10) | 33.16 (32.68–33.63) |
| **2021** | 0.32 (0.28–0.35) | 4.26 (4.12–4.40) | 34.95 (34.46–35.44) |
| **2022** | 0.32 (0.28–0.35) | 4.03 (3.89–4.17) | 33.04 (32.58–33.51) |
| **2023** | 0.28 (0.24–0.31) | 3.79 (3.65–3.92) | 30.72 (30.27–31.16) |
| **2024** | 0.32 (0.28–0.36) | 3.31 (3.20–3.44) | 32.07 (31.60–32.54) |
| **Total (1999–2024)** | 0.22 (0.20–0.24) | 3.29 (3.23–3.35) | 43.52 (42.97–44.07) |

*“Total” represents the mean age-adjusted mortality rate per 100,000 population.*

**Supplemental Table 7, Heart Failure-Related Age-Adjusted Mortality Rates per 100,000, Stratified by Urban-Rural Classification, in Patients with Myocardial Infarction in the United States, 1999 to 2020**

| **Supplemental Table 7, Heart Failure Infection-related Age-Adjusted Mortality Rates per 100,000, Stratified by Urban-Rural Classification, in Adults with Myocardial Infarction in the United States, 1999 to 2020** | | |
| --- | --- | --- |
| **Age-Adjusted Rate (95% CI)** | | |
| **Year** | **Urban** | **Rural** |
| **1999** | 14.07 (13.87 – 14.26) | 20.50 (20.02 – 20.98) |
| **2000** | 13.80 (13.61 – 14.00) | 19.86 (19.39 – 20.34) |
| **2001** | 13.07 (12.89 – 13.26) | 18.74 (18.28 – 19.19) |
| **2002** | 12.60 (12.42 – 12.78) | 17.86 (17.41 – 18.30) |
| **2003** | 11.82 (11.65 – 12.00) | 17.92 (17.48 – 18.37) |
| **2004** | 11.04 (10.87 – 11.20) | 16.91 (16.48 – 17.34) |
| **2005** | 10.57 (10.40 – 10.73) | 16.96 (16.53 – 17.39) |
| **2006** | 9.66 (9.51 – 9.81) | 15.61 (15.20 – 16.01) |
| **2007** | 9.04 (8.90 – 9.19) | 14.32 (13.94 – 14.71) |
| **2008** | 8.59 (8.45 – 8.73) | 14.28 (13.90 – 14.67) |
| **2009** | 7.77 (7.64 – 7.90) | 13.60 (13.23 – 13.98) |
| **2010** | 7.59 (7.46 – 7.72) | 12.29 (11.94 – 12.65) |
| **2011** | 7.28 (7.16 – 7.41) | 12.52 (12.17 – 12.88) |
| **2012** | 7.05 (6.92 – 7.17) | 12.20 (11.85 – 12.54) |
| **2013** | 6.90 (6.78 – 7.01) | 11.57 (11.24 – 11.91) |
| **2014** | 6.79 (6.67 – 6.90) | 11.91 (11.57 – 12.24) |
| **2015** | 6.78 (6.67 – 6.90) | 12.10 (11.76 – 12.44) |
| **2016** | 6.77 (6.65 – 6.88) | 11.78 (11.45 – 12.12) |
| **2017** | 6.84 (6.73 – 6.96) | 12.03 (11.70 – 12.37) |
| **2018** | 6.81 (6.70 – 6.92) | 11.99 (11.65 – 12.32) |
| **2019** | 6.75 (6.64 – 6.86) | 11.97 (11.64 – 12.30) |
| **2020** | 7.22 (7.11 – 7.33) | 13.10 (12.76 – 13.45) |
| **Total** | **9.04 (8.90 – 9.18)** | **14.55 (14.16 – 14.93)** |
| The data for urbanization is only available till 2020 in the CDC Wonder Database. | | |
|  |  |  |

*“Total” represents the mean age-adjusted mortality rate per 100,000 population.*

**FORECAST**

**Supplemental Table 8, Overall and Sex‐Stratified Heart Failure-Related Age-Adjusted Mortality Rates per 100,000, in Patients with Myocardial Infarction in the United States, 2025 to 2035**

| **Supplemental Table 8: Overall and Sex‐Stratified Heart Failure-Related Age-Adjusted Mortality Rates per 100,000 in Patients with Myocardial Infarction in the United States, 2025 to 2035** | | | |
| --- | --- | --- | --- |
| Year | Men, Age-Adjusted Rate (95% CI) | Women, Age-Adjusted Rate (95% CI) | Overall, Age-Adjusted Rate (95% CI) |
| 2025 | 9.55 (8.89–10.26) | 6.06 (5.83–6.30) | 8.28 (7.98–8.58) |
| 2026 | 9.38 (8.02–10.97) | 5.88 (5.65–6.12) | 8.13 (7.82–8.47) |
| 2027 | 9.22 (7.39–11.51) | 5.81 (5.58–6.04) | 8.09 (7.75–8.40) |
| 2028 | 9.06 (6.84–12.00) | 5.84 (5.59–6.12) | 8.17 (7.81–8.52) |
| 2029 | 8.90 (6.34–12.51) | 6.00 (5.72–6.30) | 8.44 (8.03–8.86) |
| 2030 | 8.75 (5.88–13.03) | 5.83 (5.54–6.12) | 8.29 (7.80–8.75) |
| 2031 | 8.60 (5.45–13.58) | 5.76 (5.44–6.10) | 8.25 (7.73–8.77) |
| 2032 | 8.45 (5.04–14.17) | 5.78 (5.42–6.20) | 8.33 (7.72–8.93) |
| 2033 | 8.30 (4.66–14.80) | 5.94 (5.54–6.42) | 8.60 (7.89–9.31) |
| 2034 | 8.16 (4.30–15.48) | 5.77 (5.32–6.33) | 8.45 (7.63–9.24) |
| 2035 | 8.02 (3.97–16.22) | 5.70 (5.22–6.34) | 8.41 (7.46–9.31) |

**Supplemental Table 9, Race‐Stratified Heart Failure-Related Age-Adjusted Mortality Rates per 100,000 in Patients with Myocardial Infarction in the United States, 2025 to 2035**

| **Supplemental Table 9, Race‐Stratified Heart Failure-Related Age-Adjusted Mortality Rates per 100,000 in Patients with Myocardial Infarction in the United States, 2025 to 2035** | | | | |
| --- | --- | --- | --- | --- |
| Year | Hispanic, Age-Adjusted Rate (95% CI) | NH American Indian or Alaska Native, Age-Adjusted Rate (95% CI) | NH Black or African American, Age-Adjusted Rate (95% CI) | NH White, Age-Adjusted Rate (95% CI) |
| 2025 | 5.55 (5.05–6.10) | 6.84 (4.47–10.48) | 8.87 (7.94–9.90) | 8.41 (8.08–8.80) |
| 2026 | 5.39 (4.50–6.46) | 6.84 (3.74–12.50) | 8.76 (7.34–10.46) | 8.27 (7.92–8.63) |
| 2027 | 5.24 (4.13–6.64) | 6.84 (3.27–14.31) | 8.65 (6.78–11.04) | 8.23 (7.89–8.60) |
| 2028 | 5.09 (3.83–6.75) | 6.84 (2.92–16.05) | 8.54 (6.25–11.68) | 8.27 (7.87–8.68) |
| 2029 | 4.94 (3.58–6.82) | 6.84 (2.64–17.74) | 8.44 (5.74–12.40) | 8.54 (8.14–8.98) |
| 2030 | 4.80 (3.36–6.86) | 6.84 (2.41–19.43) | 8.33 (5.26–13.20) | 8.40 (7.95–8.90) |
| 2031 | 4.66 (3.16–6.88) | 6.84 (2.21–21.13) | 8.23 (4.80–14.11) | 8.36 (7.85–8.92) |
| 2032 | 4.53 (2.98–6.89) | 6.84 (2.05–22.84) | 8.13 (4.36–15.14) | 8.40 (7.82–9.03) |
| 2033 | 4.40 (2.81–6.87) | 6.84 (1.90–24.57) | 8.03 (3.95–16.29) | 8.68 (8.08–9.43) |
| 2034 | 4.27 (2.66–6.85) | 6.84 (1.78–26.34) | 7.93 (3.57–17.59) | 8.54 (7.78–9.39) |
| 2035 | 4.15 (2.53–6.82) | 6.84 (1.66–28.13) | 7.83 (3.21–19.06) | 8.49 (7.63–9.47) |

**Supplemental Table 10, census‐Stratified Heart Failure-Related Age-Adjusted Mortality Rates per 100,000 in Patients with Myocardial Infarction in the United States, 2025 to 2035**

| **Supplemental Table 10, census‐Stratified Heart Failure-Related Age-Adjusted Mortality Rates per 100,000 in Patients with Myocardial Infarction in the United States, 2025 to 2035** | | | | |
| --- | --- | --- | --- | --- |
| Year | Midwest, Age-Adjusted Rate (95% CI) | Northeast, Age-Adjusted Rate (95% CI) | South, Age-Adjusted Rate (95% CI) | West, Age-Adjusted Rate (95% CI) |
| 2025 | 7.36 (6.65–8.14) | 5.38 (4.94–5.87) | 9.41 (9.05–9.83) | 8.11 (7.68–8.58) |
| 2026 | 7.12 (6.03–8.41) | 5.19 (4.59–5.87) | 9.30 (8.92–9.70) | 8.06 (7.63–8.53) |
| 2027 | 6.90 (5.46–8.72) | 5.01 (4.31–5.81) | 9.34 (8.98–9.78) | 8.06 (7.66–8.56) |
| 2028 | 6.68 (4.92–9.06) | 4.83 (4.06–5.74) | 9.54 (9.09–10.00) | 8.11 (7.67–8.56) |
| 2029 | 6.46 (4.41–9.47) | 4.66 (3.84–5.65) | 9.86 (9.36–10.40) | 8.33 (7.86–8.86) |
| 2030 | 6.26 (3.94–9.94) | 4.49 (3.64–5.55) | 9.74 (9.17–10.33) | 8.28 (7.80–8.82) |
| 2031 | 6.06 (3.50–10.47) | 4.33 (3.45–5.45) | 9.78 (9.15–10.45) | 8.27 (7.75–8.85) |
| 2032 | 5.86 (3.10–11.08) | 4.18 (3.27–5.34) | 9.99 (9.27–10.81) | 8.33 (7.72–8.99) |
| 2033 | 5.68 (2.74–11.78) | 4.03 (3.11–5.22) | 10.33 (9.44–11.33) | 8.56 (7.91–9.35) |
| 2034 | 5.50 (2.40–12.56) | 3.89 (2.96–5.11) | 10.20 (9.19–11.24) | 8.50 (7.72–9.28) |
| 2035 | 5.32 (2.10–13.46) | 3.75 (2.82–4.99) | 10.25 (9.15–11.48) | 8.50 (7.66–9.40) |

**Supplemental Table 11, Age‐Stratified Heart Failure-Related Age-Adjusted Mortality Rates per 100,000, in Patients with Myocardial Infarction in the United States, 2025 to 2035**

| **Supplemental Table 11, Age‐Stratified Heart Failure-Related Age-Adjusted Mortality Rates per 100,000, in Patients with Myocardial Infarction in the United States, 2025 to 2035** | | | |
| --- | --- | --- | --- |
| Year | Age 25-44, Age-Adjusted Rate (95% CI) | Age 45-64, Age-Adjusted Rate (95% CI) | Age 65+, Age-Adjusted Rate (95% CI) |
| 2025 | 0.29 (0.19–0.43) | 3.28 (2.94–3.67) | 34.91 (33.65–36.21) |
| 2026 | 0.27 (0.17–0.43) | 3.27 (2.72–3.92) | 34.11 (32.91–35.43) |
| 2027 | 0.26 (0.15–0.42) | 3.25 (2.58–4.11) | 33.78 (32.53–35.11) |
| 2028 | 0.25 (0.15–0.42) | 3.24 (2.48–4.24) | 33.91 (32.47–35.50) |
| 2029 | 0.24 (0.15–0.41) | 3.23 (2.42–4.32) | 34.86 (33.25–36.68) |
| 2030 | 0.24 (0.14–0.41) | 3.23 (2.38–4.38) | 34.06 (32.26–36.04) |
| 2031 | 0.24 (0.14–0.40) | 3.22 (2.35–4.42) | 33.73 (31.74–36.01) |
| 2032 | 0.24 (0.14–0.40) | 3.22 (2.33–4.45) | 33.86 (31.63–36.47) |
| 2033 | 0.24 (0.14–0.40) | 3.21 (2.31–4.47) | 34.80 (32.03–37.94) |
| 2034 | 0.24 (0.14–0.40) | 3.21 (2.30–4.48) | 34.01 (31.00–37.48) |
| 2035 | 0.24 (0.14–0.40) | 3.21 (2.29–4.49) | 33.68 (30.27–37.74) |

**Supplemental Table 12, Heart Failure-related Age-Adjusted Mortality Rates per 100,000, Stratified by Urban-Rural Classification, in Patients with Myocardial Infarction in the United States, 2025 to 2035**

| **Supplemental Table 12, Heart Failure-related Age-Adjusted Mortality Rates per 100,000, Stratified by Urban-Rural Classification, in Patients with Myocardial Infarction in the United States, 2025 to 2035** | | |
| --- | --- | --- |
| **Age-Adjusted Rate (95% CI)** | | |
| **Year** | **Urban** | **Rural** |
| **2021** | 7.02 (6.92 – 7.13) | 12.54 (12.21 – 12.88) |
| **2022** | 7.03 (6.89 – 7.18) | 12.49 (12.13 – 12.86) |
| **2023** | 7.10 (6.88 – 7.32) | 12.63 (12.22 – 13.06) |
| **2024** | 7.24 (6.90 – 7.55) | 12.97 (12.49 – 13.44) |
| **2025** | 7.23 (6.77 – 7.67) | 12.96 (12.41 – 13.64) |
| **2026** | 7.23 (6.64 – 7.85) | 12.91 (12.24 – 13.69) |
| **2027** | 7.30 (6.54 – 8.11) | 13.05 (12.16 – 14.08) |
| **2028** | 7.45 (6.52 – 8.43) | 13.40 (12.35 – 14.62) |
| **2029** | 7.43 (6.33 – 8.59) | 13.39 (12.14 – 14.88) |
| **2030** | 7.44 (6.20 – 8.85) | 13.34 (11.83 – 15.04) |
| **2031** | 7.51 (6.12 – 9.18) | 13.49 (11.81 – 15.49) |
| **2032** | 7.66 (6.08 – 9.70) | 13.85 (11.90 – 16.24) |
| **2033** | 7.64 (5.88 – 9.99) | 13.83 (11.56 – 16.51) |
| **2034** | 7.65 (5.70 – 10.34) | 13.78 (11.35 – 16.84) |
| **2035** | 7.73 (5.60 – 10.96) | 13.94 (11.18 – 17.29) |
| The data for urbanization is only available till 2020 in the CDC Wonder Database. | | |
|  |  |  |

**Supplemental Table 13, Sensitivity Analysis of Age-Adjusted Mortality Rates for Heart Failure as a Multiple Cause of Death in Patients with Myocardial Infarction 2025–2035**

| **Supplemental Table 13, Sensitivity Analysis of Age-Adjusted Mortality Rates for Heart Failure as a Multiple Cause of Death in Patients with Myocardial Infarction 2025–2035** | |
| --- | --- |
| **Year** | **Age-Adjusted Rate (95% CI)** |
| **2025** | 5.46 (5.29 – 5.62) |
| **2026** | 5.32 (5.16 – 5.49) |
| **2027** | 5.26 (5.10 – 5.43) |
| **2028** | 5.27 (5.08 – 5.46) |
| **2029** | 5.36 (5.13 – 5.61) |
| **2030** | 5.22 (4.97 – 5.47) |
| **2031** | 5.16 (4.89 – 5.47) |
| **2032** | 5.17 (4.83 – 5.53) |
| **2033** | 5.26 (4.85 – 5.69) |
| **2034** | 5.13 (4.68 – 5.59) |
| **2035** | 5.06 (4.57 – 5.63) |

#

# **Supplementary Code: Forecasting of Age-Adjusted Mortality Rates (AAMRs)**

This R script performs forecasting of annual age-adjusted mortality rates (AAMRs) separately for males, females, and the overall population.

### **Workflow**

1. Import annual AAMR data from an Excel file.
2. Split observations into:
   - Training period: 1999–2020
   - Validation period: 2021–2024
3. Fit ARIMA and Prophet models using rolling-origin validation.
4. Evaluate predictive performance using root mean squared error (RMSE).
5. Select the model with the lowest RMSE for each sex group.
6. Refit the selected model using all available observations (1999–2024).
7. Generate forecasts for 2025–2035 with 95% confidence intervals.
8. Export validation results, RMSE summaries, and future forecasts to Excel files.
9. Produce sex-specific and combined forecast figures.

### **Required Input File**

The input dataset (Excel.xlsx) must contain the following variables:

- Year – calendar year
- AAMR – age-adjusted mortality rate
- Sex – Male, Female, or Overall

### **Required R Packages**

- dplyr
- forecast
- prophet
- ggplot2
- openxlsx
- readxl
